# Impact of clinical trial participation on the survival of patients with newly diagnosed advanced ovarian cancer

**DOI:** 10.3389/fonc.2025.1591000

**Published:** 2025-05-06

**Authors:** Yong Jae Lee, Jung-Yun Lee, Eun Ji Nam, Sang Wun Kim, Sunghoon Kim, Young Tae Kim

**Affiliations:** Department of Obstetrics and Gynecology, Institute of Women’s Life Medical Science, Yonsei University College of Medicine, Seoul, Republic of Korea

**Keywords:** clinical trial, ovarian cancer, survival, standard of care (SOC), maintenance therapy

## Abstract

**Background:**

Clinical trials provide access to novel treatments that may offer survival benefits to ovarian cancer patients. This study investigates whether clinical trial participation is associated with improved survival in newly diagnosed advanced ovarian cancer.

**Methods:**

We retrospectively investigated patients treated for advanced ovarian cancer at Yonsei Cancer Hospital between 2019 and 2021. During this period, the standard of care included cytoreductive surgery with platinum-based chemotherapy, with or without bevacizumab, followed by maintenance therapy with PARP inhibitors or bevacizumab. This study included 202 patients with stage III-IV, 82 participated in clinical trials [DUO-O (Bevacizumab+immunotherapy (IO)+/- PARP inhibitors), KEYLYNK-001 (PARP inhibitors +IO), ATHENA (PARP inhibitors), TRU-D (IO+IO)] and 120 received standard-of-care.

**Result:**

The median follow-up duration was 39.8 months. Disease recurrence occurred in 123 (60.9%) patients and 45 (22.3%) patients died. Among the patients in both groups, there were no significant differences in age, histologic type, stage, median CA-125 level, comorbidities, and *BRCA1/2* status. There were also no differences in the incorporation of hyperthermic intraperitoneal chemotherapy, neoadjuvant chemotherapy, or residual disease after cytoreductive surgery. Clinical trial participation was associated with significantly improved progression-free survival (31.4 vs. 19.1 months; HR, 0.67; 95% CI, 0.46 to 0.97; p = 0.035) and overall survival (both not reached; HR, 0.54; 95% CI, 0.31 to 0.93; p = 0.024) compared to standard of care.

**Conclusion:**

Clinical trial participation was associated with improved survival compared with standard of care in patients with newly diagnosed advanced ovarian cancer.

## 1 Introduction

Ovarian cancer is the most lethal gynecologic malignancy in women (1). Cytoreductive surgery with platinum-doublet chemotherapy is the standard treatment for advanced ovarian cancer (2, 3). Despite primary treatment, most patients experience relapse and become resistant to platinum based chemotherapy (4, 5). Patients with platinum-resistant recurrent ovarian cancer have a poor prognosis, and the available treatment options are very limited (6, 7). Therefore, there is an unmet need to develop novel therapeutic strategies to improve survival outcomes for patients with advanced ovarian cancer.

Recently, several randomized controlled trials reported that poly (ADP-ribose) polymerase (PARP) inhibitors maintenance therapy significantly improves survival outcomes in newly diagnosed advanced ovarian cancer, depending on the expression of *BRCA* mutation or homologous recombination deficiency (8–10). The introduction of PARP inhibitors has led to a new era of treatment in newly diagnosed advanced ovarian cancer. Therefore, clinical trials are crucial for evaluating new therapeutic options, establishing new standards of oncologic care and enhancing survival outcomes. The National Comprehensive Cancer Network Guidelines for oncology and the American Society of Clinical Oncology strongly recommend clinical trial enrollment (11, 12). Participation in clinical trials is considered beneficial for patients with newly diagnosed advanced ovarian cancer. However, it remains unclear whether participation in clinical trials is associated with improved survival outcomes. In this study, we aimed to investigate whether clinical trial participation is associated with improved survival outcomes in patients with newly diagnosed advanced ovarian cancer.

## 2 Materials and methods

This single, retrospective cohort study was conducted on patients with newly diagnosed advanced (stage III or IV) epithelial ovarian, fallopian tube, and/or primary peritoneal carcnoma who were treated at Yonsei Cancer Hospital between 2019 and 2021. All patients underwent primary debulking surgery followed by platinum-based chemotherapy or neoadjuvant chemotherapy followed by interval cytoreductive surgery. Patients were stratified into two cohorts. 1) Clinical trial cohort: Patients who participated in clinical trials were eligible for inclusion if they had completed standard chemotherapy or chemotherapy in combination with ICIs, and subsequently received at least one cycle of maintenance therapy as per protocol. Details of the clinical trials in which the patients were enrolled are shown in **Supplementary Table S1**) Standard of care cohort: Patients with no clinical trial participation. During this period, the standard of care included cytoreductive surgery combined with platinum-based chemotherapy, with or without bevacizumab, followed by maintenance therapy with PARP inhibitors for patients with *BRCA* mutations and bevacizumab for those with *BRCA* wild-type. Patients who received at least one cycle of treatment in any clinical trial protocol were included in the clinical trial cohort. Recurrence was defined as the date of appearance of radiologically detected disease during a follow-up examination. A rise in serum cancer antigen (CA-125) without clinical signs of relapse was not counted as progression but generally triggered further radiological examinations. During follow-up, CA-125 and imaging studies were performed every 3 months for the first 2 years after treatment and every 6 months thereafter.

The following data were extracted from the patients’ medical records: age, pretreatment CA-125 levels, International Federation of Gynecology and Obstetrics (FIGO) stage, histology, *BRCA* status, medical comorbidities, residual disease after cytoreductive surgery, treatment with PARP inhibitors, bevacizumab, immune checkpoint inhibitor or hyperthermic intraperitoneal chemotherapy (HIPEC), chemotherapy regimens, total cycles of chemotherapy, date of progression or recurrence, and date of last follow-up.

Demographic data were summarized as the median (range) or frequency (percentage). The chi-squared and Fisher exact tests were used to compare the study variables. Progression-free survival and overall survival were analyzed with the Kaplan-Meier method and log-rank test. Factors identified as significant in the univariate analyses were subjected to multivariate analysis. Cox regression analysis was used to evaluate the effects of the prognostic factors, expressed as hazard ratios (HRs) with 95% confidence intervals (CIs). These included age, FIGO stage, BRCA mutation status, presence of medical comorbidities, residual disease status after cytoreductive surgery, use of neoadjuvant chemotherapy, total number of chemotherapy cycles, use of HIPEC, administration of maintenance therapy. Variables were included as categorical or continuous as appropriate. For all analyses, P < 0.05 was considered statistically significant.

## 3 Results

Of 202 patients with newly diagnosed advanced ovarian cancer who were treated between 2019 and 2021, 82 (40.6%) patients were treated in a clinical trial protocol, and 120 (59.4%) were treated with standard of care. The patient and clinical characteristics are shown in **Table 1**. There were no significant differences in age (p = 0.755), histologic type (p = 0.469), stage (p = 0.283), median CA-125 level (p = 0.281), or medical comorbidities including hypertension (p = 0.070), and diabetes mellitus (p = 0.686) between patients treated with standard of care compared with those treated in clinical trials. Additionally, *BRCA* status (p = 0.987) was similar between the groups.

**Table 1:** Baseline patients’ characteristics.

Variable	Clinical trial (n = 82)	SOC (n = 120)	*P*
Median age, years (range)	56 (34-81)	59 (35-83)	0.755
Histologic type, n (%)			
High grade serous	76 (92.7%)	107 (89.2%)	0.469
Other^a^	6 (7.3%)	13 (10.8%)	
FIGO stage, n (%)			
III	30 (36.6%)	53 (44.2%)	0.283
IV	52 (63.4%)	67 (55.8%)	
Median CA-125 level, U/mL (range)	987.8 (13.3-23844.6)	690.0 (11.5-25000)	0.281
BRCA status, n (%)			
Wild-type	55 (67.1%)	77 (64.2%)	0.987
BRCA1/2 mutation	22 (26.8%)	36 (30.0%)	
Unknown	5 (6.1%)	7 (5.8%)	
Medical comorbidities			
HTN	18 (22.0%)	36 (30.0%)	0.070
DM	8 (9.8%)	16 (13.3%)	0.686
Other^b^	10 (12.2%)	17 (14.2%)	0.834
Residual disease, n (%)			
No gross tumor	60 (73.2%)	79 (65.8%)	0.401
Optimal (≤1.0 cm)	19 (23.2%)	32 (26.7%)	
Suboptimal	3 (3.7%)	9 (7.5%)	
Neoadjuvant chemotherapy, n (%)			
No	39 (47.6%)	57 (47.5%)	0.553
Yes	43 (52.4%)	63 (52.5%)	
HIPEC, n (%)			
No	75 (91.5%)	112 (93.3%)	0.406
Yes	7 (8.5%)	8 (6.7%)	
Maintenance therapy ^c^, n (%)			
No	0 (0%)	77 (64.2%)	< 0.001
Yes	82 (100%)	43 (35.8%)	
Cycles of total chemotherapy, median (range)	6 (6-8)	6 (1-12)	0.001

FIGO, International Federation of Gynecology and Obstetrics; HIPEC, hyperthermic intraperitoneal chemotherapy.

a Clear cell, endometrioid, carcinosarcoma, mixed carcinoma.

b Atrial fibrillation, arrhythmia, asthma, coronary artery occulusive disease, hepatitis B, hyperthyroidism, hypothyroidism, systemic lupus erythematosus.

c PARP inhibitor, immune checkpoint inhibitor, bevacizumab.

Regarding oncologic outcomes, there were no significant differences between the standard of care group and the clinical trial group in terms of residual disease after initial debulking surgery (p = 0.401), receipt of neoadjuvant chemotherapy (p = 0.553), or receipt of HIPEC (p = 0.406). Maintenance therapy was significantly higher in the clinical trial group (100%) compared to the standard of care group (35.8%) (p < 0.001). Additionally, the median number of chemotherapy cycles was significantly different between the groups, with the clinical trial group receiving a median of 6 cycles (range: 6-8) and the standard of care group receiving a median of 6 cycles (range: 1-12) (p = 0.001).

**Figure 1:**
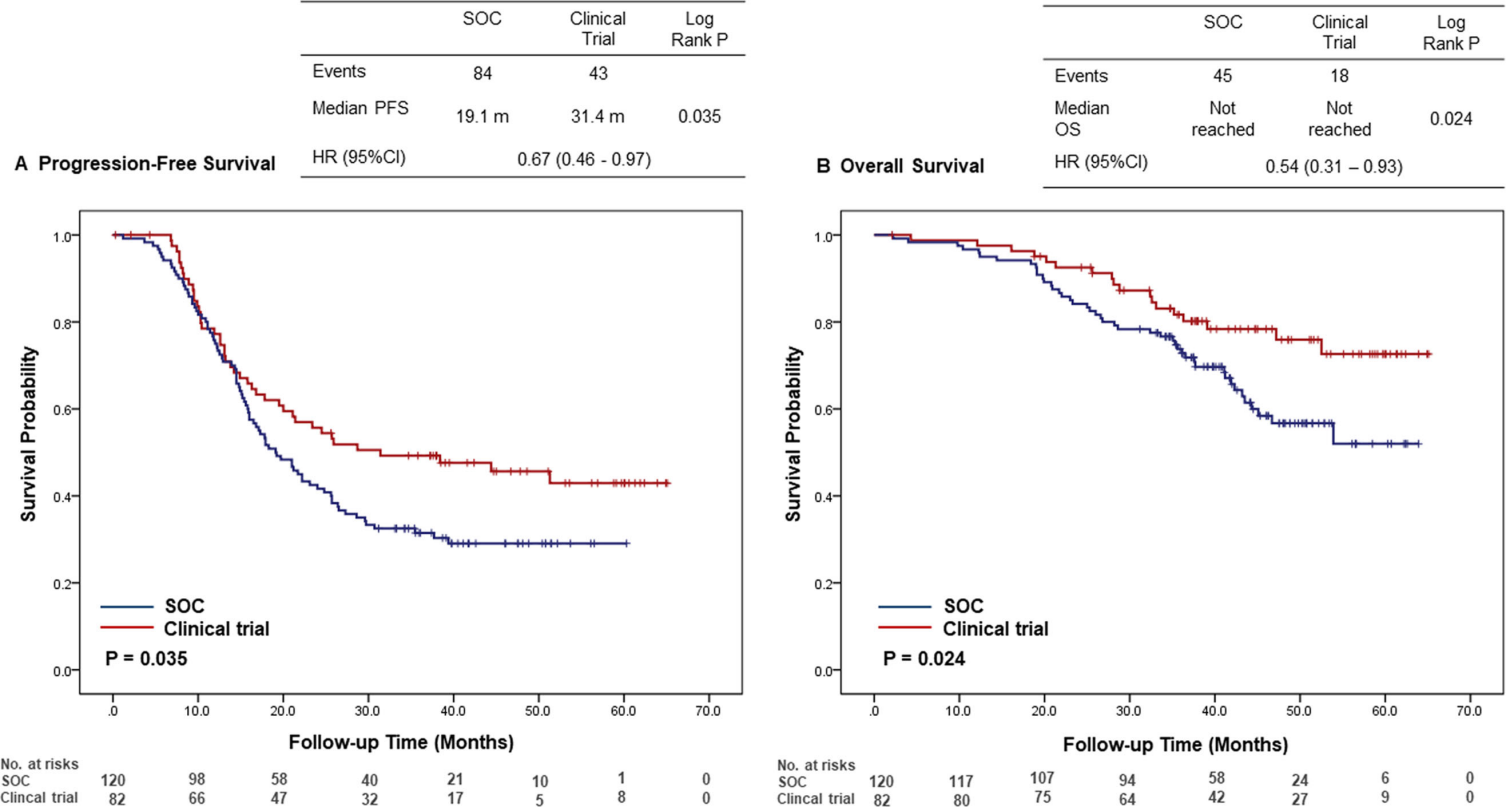
Kaplan-Meier curves for **(A)** progression-free survival and **(B)** overall survival comparing clinical trial participants and patients receiving standard of care. Survival distributions were compared using the log-rank test, and hazard ratios with 95% confidence intervals (CIs) were calculated using the Cox proportional hazards model. The number of patients at risk at each time point is displayed below the X-axis. CI, confidence intervals; HR, hazard ratio; OS, overall survival; PFS, progression-free survival; SOC, standard of care.

At the time of analysis, the duration of median follow-up was 39.8 months (2.1-65.1 months). Progression occurred in 127 patients (62.9%) overall, with 43 of 82 patients (52.4%) in the clinical trial group and 84 of 120 patients (70.0%) in the standard of care group. The median progression-free survival was 31.4 months in the clinical trial group and 19.1 months in the standard of care group (HR, 0.67; 95% CI, 0.46–0.97; p = 0.035) (**Figure 1A**). Eighteen of 82 patients (22.0%) in the clinical trial group and 45 of 120 (37.5%) patients in the standard of care group died. The median overall survival was not reached in both groups (HR, 0.54; 95% CI, 0.31–0.93; p = 0.024) (**Figure 1B**). Median follow-up was 40.9 months in the clinical trial group and 40.1 months in the standard-of-care (SOC) group. The 2-year and 3-year overall survival rates were 92.5% (95% CI, 86.8–98.2%) and 81.7% (95% CI, 73.1–90.3%), respectively, in the clinical trial group. In the SOC group, the 2-year and 3-year OS rates were 84.2% (95% CI, 77.7–90.7%) and 73.8% (95% CI, 65.8–81.8%), respectively.

The results of the multivariate Cox regression analyses of progression-free survival and overall survival in all patients are shown in **Table 2**. On multivariate analysis, clinical trial participation was associated with improved progression-free survival compared with standard of care (HR, 0.66; 95% CI, 0.31–0.94). For overall survival, multivariate analysis showed that clinical trial participation was an independent prognostic factor (HR, 0.55; 95% CI, 0.32–0.96). *BRCA1/2* mutation was an independent prognostic factor associated with a lower risk of progression (HR, 0.39; 95% CI, 0.16–0.94) and death (HR, 0.24; 95% CI, 0.07–0.81) in clinical trial participation group. Any residual disease was significantly associated with a higher risk of progression (HR, 2.10; 95% CI, 1.53–3.26) and death (HR, 2.21; 95% CI, 1.27–3.22) in clinical trial participation group.

**Table 2:** Multivariate analyses for progression-free and overall survival using a Cox proportional hazards model with categorical variables.

Variables	PFS		OS	
	HR (95% CI)	*P*	HR (95% CI)	*P*
Age				
≤58	Ref		Ref	
>58	1.00 (0.98-1.02)	0.908	1.02 (0.99-1.05)	0.195
FIGO stage				
III	Ref		Ref	
IV	0.88 (0.58-1.32)	0.543	0.78 (0.43-1.41)	0.408
*BRCA* status				
Wild-type	Ref		Ref	
*BRCA1/2* mutation	0.39 (0.16-0.94)	0.036	0.24 (0.07-0.81)	0.022
Medical comorbidities				
No	Ref		Ref	
Any comorbidities	1.02 (0.68-1.47)	0.685	1.33 (0.72-2.08)	0.565
Residual disease				
No	Ref		Ref	
Any residual	2.10 (1.53-3.26)	0.018	2.21 (1.27-3.22)	0.026
Neoadjuvant chemotherapy				
No	Ref		Ref	
Yes	1.01 (0.68-1.58)	0.085	6.66 (0.91-48.70)	0.062
HIPEC				
No	Ref		Ref	
Yes	1.92 (0.91-4.05)	0.088	6.66 (0.91-48.70)	0.062
Maintenance therapy				
No	Ref		Ref	
Yes	1.00 (0.60-1.69)	0.989	0.75 (0.38-1.47)	0.398
Cycles of total chemotherapy				
≤6	Ref		Ref	
>6	0.82 (0.55-1.21)	0.318	0.62 (0.21-1.82)	0.620
Clinical trial				
SOC	Ref		Ref	
Clinical trial	0.66 (0.31-0.94)	0.028	0.55 (0.32-0.96)	0.034

HR, hazard ratio; CI, confidence interval; FIGO, International Federation of Gynecology and Obstetrics; HIPEC, hyperthermic intraperitoneal chemotherapy; SOC, standard of care.

## 4 Discussion

In this study, we investigated whether participation in clinical trials is associated with improved survival outcomes in patients with newly diagnosed advanced ovarian cancer. The results demonstrated that clinical trial participation significantly improved progression-free survival and overall survival compared to standard of care.

Previous studies have shown the role of participation in clinical trials on survival outcomes in ovarian cancer. In front-line setting, Khoja et al. (13) evaluated the clinical trial effect in patients treated with three first-line clinical trials (ICON-5, ICON-7, and SCOTROC-4). There was no significant difference in survival outcomes compared with patients treated with standard of care. Robinson et al. (14) showed that participation in clinical trials was associated with improved survival. In the recurrent setting, Morton et al. (15) evaluated whether participation in clinical trials was associated with oncologic outcomes in patients with platinum-resistant ovarian cancer. Participation in clinical trial was associated with improved overall survival compared with standard of care. Nitecki et al. (16) reported the association between clinical trial enrollment and aggressive care at the end of life. Clinical trial enrollment was significantly associated with an improvement in overall survival. These data showed that participation in clinical trials has been shown associated with improved survival in platinum-resistant ovarian cancer. However, the contribution of participation in clinical trials to improved survival remains a subject of debate.

Recent advancements in the treatment of newly diagnosed advanced ovarian cancer have been driven by extensive clinical trials, leading to a paradigm shift in clinical practice. Historically, maximal cytoreductive surgery combined with platinum-based chemotherapy was the standard of care. However, several clinical trials have shown that integrating novel therapies can substantially improve patient outcomes. The incorporation of bevacizumab, an antiangiogenic agent, has been shown to significantly improve progression-free survival in patients with advanced-stage ovarian cancer (17, 18). Furthermore, maintenance therapies with PARP inhibitors such as olaparib and niraparib have markedly improved overall survival in these patients (8, 9).

In our study, we included patients who participated in clinical trials that combined PARP inhibitors, bevacizumab, and immunotherapy aimed at achieving long-term remission and improving the cure rate in front-line treatment. Although the clinical trial cohort involved heterogeneous treatment protocols, the consistent survival benefit suggests a shared effect of intensified therapeutic strategies. These included PARP inhibitors, immune checkpoint inhibitors (ICIs), and anti-angiogenic agents, administered alone or in combination. Mechanistically, PARP inhibitors increase tumor immunogenicity via DNA damage accumulation, ICIs restore antitumor immune surveillance, and bevacizumab enhances immune infiltration by normalizing tumor vasculature. Dual checkpoint blockade may further activate immune responses in immunologically “cold” tumors. Notably, all clinical trial participants received maintenance therapy regardless of biomarker status, in contrast to the limited use in the standard-of-care group. Access to these novel agents and broader maintenance application likely contributed to the improved progression-free and overall survival observed.

This study highlights the association between clinical trial enrollment and survival outcomes in patients with newly diagnosed advanced ovarian cancer. Notably, participation in clinical trials was associated with improved progression-free survival and overall survival compared to standard of care. These findings suggest the importance of encouraging clinical trial enrollment for patients with newly diagnosed advanced ovarian cancer, as it provides access to novel therapies that may offer superior survival benefits. A limitation of our study is its retrospective nature and reliance on medical records, as well as being based on the experience of a single institution. Second, the potential for selection bias cannot be disregarded as a factor in the observed improved outcomes. Patients enrolled in clinical trials are selected based on stringent criteria such as performance status, age, and comorbidities as outlined in the study protocols. However, there may be subtle variations among participants that are not easily captured by standard inclusion criteria but can be identified by experienced clinicians. Identifying such differences could help determine which patients are more likely to respond to the treatment and tolerate it better, ultimately resulting in longer survival. Third, the potential influence of the Hawthorne effect should be considered. Patients participating in clinical trials may experience improved outcomes not solely due to the investigational treatment itself, but also due to increased clinical attention, structured follow-up, and enhanced patient engagement. Although this effect cannot be quantified in our study, it may partially explain the observed differences in survival outcomes between the two cohorts (19). Globally, clinical trial participation among ovarian cancer patients remains low, with rates under 10% (20, 21). Nitecki et al. reported that only 2.6% of patients with ovarian cancer were enrolled in therapeutic clinical trials, despite national guidelines emphasizing clinical trial consideration throughout treatment. These low participation rates highlight a significant gap between the development of novel therapeutics and their implementation in clinical practice. These findings suggest that improving access to clinical trials could potentially enhance real-world survival outcomes.

In conclusion, our study demonstrates that participation in clinical trials is associated with significantly improved survival outcomes in patients with newly diagnosed advanced ovarian cancer. These findings support the encouragement of clinical trial enrollment for patients with newly diagnosed advanced ovarian cancer, as clinical trials provide access to novel treatments that may offer superior survival benefits compared to standard of care.

## Data Availability

The raw data supporting the conclusions of this article will be made available by the authors, without undue reservation.
