# Supplementary material for: Impact of clinical trial participation on the survival of patients with newly diagnosed advanced ovarian cancer
Source: Front Oncol. 2025 May 6;15:1591000. doi: 10.3389/fonc.2025.1591000 (PMC12088932; doi:10.3389/fonc.2025.1591000)
Supplement: Supplementary file 1 [file Table1.docx]

Supplementary Table 1. Clinical trial details

| ClinicalTrials.gov identifier | Investigational agents | N (%) | Phase |
| --- | --- | --- | --- |
| DUO-O  (NCT03737643) | Chemotherapy + Durvalumab + Bevacizumab followed by Durvalumab, Bevacizumab, olaparib maintenance | 19 (23.1%) | III |
| KEYLYNK-001  (NCT03740165) | Chemotherapy + Pembrolizumab  followed by olaparib maintenance | 18 (22.0%) | III |
| ATHENA  (NCT03522246) | Rucaparib maintenance | 14 (17.1%) | III |
| TRU-D  (NCT03899610) | Durvalumab + Tremelimumab + Chemotherapy | 31 (37.8%) | II |
